# Predictive effect of different blood lipid parameters combined with carotid intima-media thickness on coronary artery disease

**DOI:** 10.3389/fcvm.2022.1105413

**Published:** 2023-01-12

**Authors:** Bingyan Yu, Ying Wu, Wei Li, Langping Zhou, Yan Lin, Weimian Wang, Guang Li, Yingling Zhou, Xiangming Hu, Xiaohong Li

**Affiliations:** ^1^School of Medicine, South China University of Technology, Guangzhou, China; ^2^Department of Cardiology, Guangdong Provincial People’s Hospital (Guangdong Academy of Medical Sciences), Southern Medical University, Guangzhou, China; ^3^Department of Cardiology, Guangdong Provincial People’s Hospital Zhuhai Hospital (Zhuhai Golden Bay Center Hospital), Zhuhai, China; ^4^Medical Research Center, Guangdong Provincial Key Laboratory of South China Structural Heart Disease, Guangdong Provincial People’s Hospital (Guangdong Academy of Medical Sciences), Southern Medical University, Guangzhou, China

**Keywords:** lipoprotein (a), blood lipid indices, carotid intima-media thickness, carotid ultrasound, coronary artery disease

## Abstract

**Background:**

Blood lipids disorder and atherosclerosis are closely related to coronary artery disease (CAD). This study aims to compare different blood lipid parameters combined with carotid intima-media thickness (cIMT) in predicting CAD.

**Methods:**

This was a retrospective study including patients who underwent coronary angiography for highly suspected CAD. Blood samples were taken for lipid profile analysis and cIMT was evaluated by carotid ultrasound. Logistic analysis was used to establish different models of different lipid parameters in predicting CAD. The area under the receiver operating characteristic curve (AUC) was used to examine the predictive value. The optimal lipid parameter was also used to explore the relationship with multi-vessel CAD.

**Results:**

Patients were classified into two groups based on whether CAD existed. Compared with non-CAD patients, the CAD group had higher lipoprotein (a) [Lp (a)], apolipoprotein B/apolipoprotein A, total cholesterol/high-density lipoprotein cholesterol (HDL-C), triglyceride/HDL-C and LDL-C/HDL-C. According to the AUCs, Lp (a) combined with cIMT (AUC: 0.713, *P* < 0.001) had the best performance in predicting CAD compared to other lipid parameters. High level of Lp (a) was also associated with multi-vessel CAD (odds ratio: 1.41, 95% confidence interval: 1.02–1.95, *P* = 0.036).

**Conclusion:**

For patients with highly suspected CAD, Lp (a) better improved the predictive value of CAD rather than most of blood lipid indices, especially in the absence of high levels of LDL-C. Lp (a) also can be used to predict the multi-vessel CAD.

## Introduction

Although the treatment of coronary artery disease (CAD) has greatly improved in recent years, the incidence and mortality of CAD remains high (1). Atherosclerosis, as the main pathophysiological mechanism of CAD, closely related to lipid disorders that is poor-controlled (2). Early screening of CAD based on atherosclerosis is of great importance, and there is an urgent need to predict CAD using clinically available information.

The role of lipid parameters in CAD is controversial. Previous studies have shown that blood lipid indices, such as triglyceride/high-density lipoprotein cholesterol (TG/HDL-C) ratio, apolipoprotein B/apolipoprotein A-I (Apo B/Apo A-I) ratio, total cholesterol (TC)/HDL-C ratio or low-density lipoprotein cholesterol (LDL-C)/HDL-C have a better predictive value of CAD than a single parameter (3–8). Recent findings showed that blood lipid indices were significantly correlated with the risk of intracranial atherosclerotic stenosis and metabolic syndrome (9, 10). The combination of lipid parameters represents relative status of blood lipids, which can comprehensively reflect the balance between atherosclerosis and anti-atherosclerosis and accurately assess lipid deposition (11, 12). Lipoprotein (a) [Lp (a)], which has LDL-like moiety comprising Apo B and apolipoprotein (a) [Apo (a)], is independently associated with the diagnosis and prognosis of CAD (13). In an Asian population study, Lp (a) concentration was a risk factor for CAD, and higher levels of Lp (a) increased the risk of CAD (14). As a marker of the residual risk of cardiovascular disease, Lp (a) has a stronger correlation with CAD than LDL-C when LDL-C is in low levels (15, 16). However, it is still unknown whether Lp (a) is superior than the new lipid indices in predicting CAD.

Carotid intima-media thickness (cIMT), a widely used non-invasive marker of subclinical atherosclerosis, can also be used as a preliminary screening tool for CAD (17–19). However, a meta-analysis suggested that the sensitivity and specificity of cIMT for the diagnosis of CAD were quite low, indicating that using cIMT alone as CAD screening tool was still insufficient (20). Since the total load of atherosclerosis is closely related to the concentration of blood lipids, morphological assessment of atherosclerosis based on cIMT has certain limitations in predicting CAD (21–24). Considering subclinical atherosclerosis status and blood lipid profiles together would effectively improve the accuracy of the diagnosis of CAD.

In clinical screening for CAD, blood lipid parameters and cIMT are considered to be available tools. However, it is still unknown which blood lipid parameters combined with cIMT has the optimal accuracy in predicting CAD. Therefore, this study was aimed to compare the predictive value of different blood lipid parameters combined with cIMT on CAD.

## Materials and methods

### Study design and participants

This retrospective study included 1,598 consecutive patients who underwent coronary angiography for highly suspected CAD, including chest pain with typical change in ECG or severe lesion in coronary CT angiography, from September 2014 to July 2015 in Guangdong Provincial People’s Hospital. A total of 105 patients with a history of stroke, 24 patients without cIMT measurement, and 74 patients with missing information on blood lipid parameters were excluded.

This study was approved by the Ethics Committee of Guangdong Provincial People’s Hospital, and informed verbal consent was obtained from all patients. This study was conducted in accordance with the Declaration of Helsinki.

### Data collection and measurements

Demographic data, laboratory test results, and carotid ultrasonography and coronary angiography results from the electronic medical records were collected.

The measurement of cIMT by carotid ultrasonography was detailed in our previous study (25). Carotid artery ultrasound was performed by experienced sonographers using the GE Vivid E95 (GE Healthcare, Milwaukee, WI, USA) interfaced with a 7.5–12 MHz phased array probe. The ultrasound probe scanned the entire length of the carotid artery longitudinally (from the bottom of the neck to the angle of the mandible). The region of interest for cIMT measurement is located at the far wall of the bilateral carotid arteries proximal to the bifurcation, along with ≥ 10 mm of plaque-free lesions on each side. Mean cIMT was calculated as the mean of two sides of cIMT. Two sonographers performed the cIMT measurements and determined the final results together. If there was any discrepancy, the results were determined together with a third sonographer. All patients underwent coronary angiography, and the degree of coronary stenosis was judged by two experienced cardiologists. Hypertension was diagnosed according to the European Society of Cardiology guidelines (26). Diabetes mellitus (DM) was defined based on the presence of diabetes or was diagnosed during hospitalization following the criteria of the European Society of Cardiology guidelines (27). Chronic kidney disease (CKD) was defined as previous medical history. Smoking was defined as previous or current smoking. Alcohol consumption was defined as previous drinking habit. Lp (a) was measured by AU5800 spectrophotometer (Beckman Coulter, USA) *via* immunoturbidimetry, with trihydroxy aminomethane buffer and anti Lp (a) antibody sensitized granules. HDL-C, LDL-C, TC, TG, Apo A-I, and Apo B were also detected using AU5800 spectrophotometer (Beckman Coulter, USA) *via* colorimetry or immunoturbidimetry. Based on previous studies (3–10), we selected six lipid ratios, Apo B/Apo A-I, LDL-C/Apo B, TC/HDL-C, TG/HDL-C, LDL-C/HDL-C, and HDL-C/Apo A-I, that may be associated with CAD.

### Definitions

As suggested by the American College of Cardiology in 2016, a ≥ 70% luminal diameter narrowing of an epicardial stenosis or ≥ 50% luminal diameter narrowing of the left main artery observed by visual assessment was considered as severe lesion that used as the diagnostic criteria for CAD (28). Two or more coronary arteries with severe stenosis were defined as multi-vessel CAD.

### Statistical analysis

The total procedure of statistical analysis was divided into four steps. First, Student’s *t*-test was used for normally distributed data, the Mann–Whitney U test was used for non-normally distributed data, and the Chi-square test or Fisher’s exact test was used for categorical variables to identify significant differences between two groups. Second, except for blood lipid parameters, the basic prediction model considered potential confounding factors that were both clinically and statistically significant in a backward stepwise logistic regression model (with 0.1 significance level for removal), and odds ratios (ORs) with 95% confidence intervals (CIs) were calculated. Third, a receiver operating characteristic (ROC) curve was constructed to evaluate the sensitivity, specificity, and area under the ROC curve (AUC) of different lipid parameters in predicting CAD based on the basic prediction model. Furthermore, net reclassification improvement (NRI) and integrated discrimination improvement (IDI) were calculated to evaluate the improvement of the new model when compared with the basic prediction model at low (< 50%)/intermediate (50–80%)/high risk (> 80%) of CAD. The 95% CI of NRI was obtained after bootstrapping 10,000 times. Fourth, a fully adjusted logistic model was used to explore the relationship between the optimal blood lipid parameter and multi-vessel CAD in the subgroup of diagnosed CAD. Comparisons with *P* < 0.05 (two-sided) were considered to be statistically significant. All of the analyses were performed with Stata 15.0 (StataCorp LLC, College Station, TX, USA), R version 3.4.3 (The R Project for Statistical Computing, Vienna, Austria), and EmpowerStats (X&Y Solutions, Inc., Boston, MA, USA).

## Results

### Study population

**FIGURE 1:**
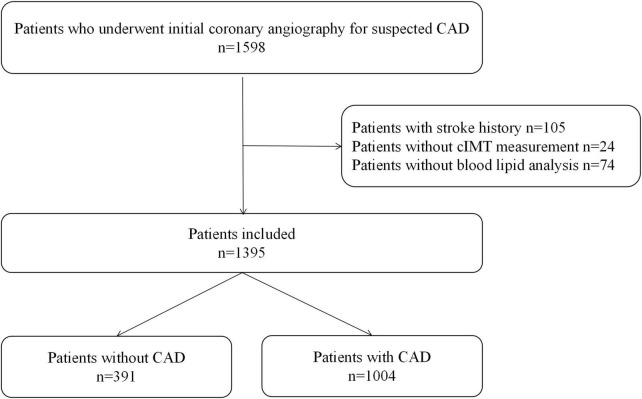
Study flowchart.

*A* total of 1,395 patients were eventually enrolled in this study (Figure 1). Baseline information of patients with and without CAD is presented in Table 1. There was no significant difference in LDL-C between the two groups. Hypertension, DM, and CKD were more prevalent in the CAD group. As for blood lipid indices, TC/HDL-C, LDL-C/HDL-C, Apo B/Apo A-I, and TG/HDL-C ratios were higher, whereas the LDL-C/Apo B ratio was lower in the CAD group. Lp (a) and mean-cIMT were also significantly higher in patients with CAD than in those without CAD. In addition, for medication history, statins was more commonly used in the CAD group.

**TABLE 1:** Baseline information of CAD and non-CAD patients.

	Non-CAD *n* = 391	CAD *n* = 1004	*P*-value
Age, year	62 ± 11	63 ± 11	0.009
Male sex	222 (56.78%)	757 (75.40%)	<0.001
Hypertension	204 (52.17%)	615 (61.25%)	0.002
Diabetes	97 (24.81%)	382 (38.05%)	<0.001
Smoking	107 (27.37%)	378 (37.65%)	<0.001
Drinking	26 (6.65%)	56 (5.58%)	0.445
CKD	45 (11.51%)	179 (17.83%)	0.004
TC, mmol/L	4.61 ± 1.18	4.43 ± 1.25	0.019
TG, mmol/L	1.55 ± 1.13	1.65 ± 1.23	0.161
LDL-C, mmol/L	2.68 ± 1.05	2.60 ± 1.07	0.245
HDL-C, mmol/L	1.15 ± 0.28	1.05 ± 0.27	<0.001
Apo A-I, mmol/L	1.28 ± 0.31	1.17 ± 0.29	<0.001
Apo B, mmol/L	0.78 ± 0.21	0.79 ± 0.24	0.448
Apo B/Apo A-I	0.61 (0.48–0.77)	0.68 (0.53–0.86)	<0.001
LDL-C/Apo B	3.35 (2.98–3.75)	3.22 (2.90–3.59)	<0.001
TC/HDL-C	4.00 (3.34–4.77)	4.15 (3.48–5.04)	0.002
TG/HDL-C	1.15 (0.80–1.76)	1.41 (0.92–2.06)	<0.001
LDL-C/HDL-C	2.21 (1.74–2.97)	2.42 (1.82–3.16)	0.007
HDL-C/Apo A-I	0.91 (0.82–0.99)	0.90 (0.82–0.98)	0.361
Mean-cIMT, mm	0.90 (0.75–1.00)	1.00 (0.85–1.10)	<0.001
Lipoprotein (a), mg/L	113.72 (70.48–215.14)	180.80 (93.34–395.25)	<0.001
Medication history			
ACEI/ARB	57 (14.58%)	171 (17.03%)	0.266
β-blocker	56 (14.32%)	185 (18.43%)	0.069
CCB	41 (10.49%)	149 (14.84%)	0.033
Diuretic	17 (4.35%)	36 (3.59%)	0.504
Statin	66 (16.88%)	432 (43.03%)	<0.001

Data are expressed as mean ± standard deviation or median (Q1–Q3) for continuous variables and *n* (%) for categorical variables.

CAD, coronary artery disease; DM, diabetes mellitus; CKD, chronic kidney disease; TC: total cholesterol; TG, triglyceride; LDL-C, low-density lipoprotein cholesterol; HDL-C, high-density lipoprotein cholesterol; Apo A-I, apolipoprotein A-I; Apo B, apolipoprotein B; cIMT, carotid intima-media thickness; ACEI, angiotensin converting enzyme inhibitor; ARB, angiotensin receptor blocker; CCB, calcium calcium entry blocker.

### Univariate and multivariate analysis

Univariate and stepwise multivariate logistic models were performed to select both clinically and statistically significant risk factors (Table 2). Age, sex, hypertension, smoking, alcohol consumption, DM, and mean-cIMT were associated with CAD and included in the basic prediction model. Among these, mean-cIMT was the strongly associated with the presence of CAD (OR 2.73, 95% CI: 1.64–4.52, *P* < 0.001).

**TABLE 2:** Backward stepwise logistic analysis.

Variable	Univariate		Multivariate	
	OR (95% CI)	*P*-value	OR (95% CI)	*P*-value
Age	1.01 (1.00–1.03)	0.009	1.01 (1.00–1.03)	0.024
Male sex	2.33 (1.82–2.98)	<0.001	2.41 (1.82–3.19)	<0.001
Hypertension	1.45 (1.15–1.83)	0.002	1.36 (1.05–1.75)	0.018
Smoking	1.60 (1.24–2.07)	0.000	1.35 (1.00–1.82)	0.052
Alcohol consumption	0.83 (0.51–1.34)	0.445	0.57 (0.34–0.96)	0.035
CKD	1.67 (1.18–2.37)	0.004	–	–
DM	1.86 (1.43–2.42)	<0.001	1.75 (1.33–2.30)	<0.001
Mean-cIMT	3.63 (2.25–5.88)	<0.001	2.73 (1.64–4.52)	<0.001

CKD, chronic kidney disease; DM, diabetes mellitus; cIMT, carotid intima-media thickness; OR, odds ratio; CI, confidence interval.

### Predictive value of different blood lipid parameters on CAD

**FIGURE 2:**
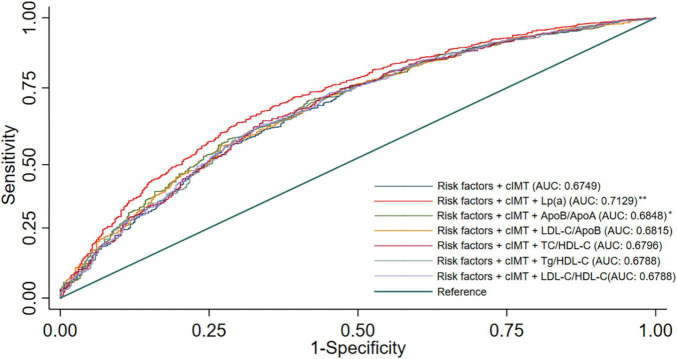
Receiver operating characteristic curves for models with different lipid parameters. Illustration: Basic prediction model adjusted for age, sex, hypertension, smoking, alcohol consumption, DM, and mean-cIMT; ^*^P < 0.05 compared with basic prediction model; ^**^P < 0.001 compared with basic prediction model.

The ROC curves of the different blood lipid parameters on the basic prediction model for predicting CAD are shown in Figure 2. The addition of Lp (a) as well as Apo B/Apo A-I ratio improved the prediction effect of the basic prediction model [AUC: 0.7129 for Lp (a), *P* < 0.001; AUC: 0.6848 for Apo B/Apo A-I ratio, *P* < 0.05]. However, when LDL-C/Apo B, TC/HDL-C, TG/HDL-C, and LDL-C/HDL-C ratios were added into the basic prediction model, the AUC of the new model was not significantly improved.

The new model that included Lp (a) achieved an NRI of 12.8%, as compared with the basic prediction model for predicting CAD (Table 3), which means that 12.8% of patients were correctly reclassified. The IDI is also listed in Table 3 and also showed a significant improvement in accuracy generated with the new model. These results indicated that the new model including Lp (a) had better predictive capability for CAD than the other blood lipid indices.

**TABLE 3:** Comparison of different lipid parameters for predicting CAD based on basic prediction model using the NRI and IDI with cut-off point at low (< 50%)/intermediate (50–80%)/high risk (> 80%) of CAD.

	Lp (a)	Apo B/Apo A-I	LDL-C/Apo B	TG/HDL-C	TC/HDL-C	LDL-C/HDL-C
NRI	0.126** (0.051 to 0.224)	0.028 (−0.014 to 0.113)	0.040* (−0.014 to 0.105)	0.010 (−0.019 to 0.094)	0.005 (−0.024 to 0.086)	0.012 (−0.023 to 0.071)
IDI	0.032** (0.024 to 0.040)	0.008* (0.003 to 0.012)	0.005* (0.000 to 0.009)	0.004* (0.000 to 0.008)	0.005* (0.00 to 0.008)	0.003* (0.000 to 0.006)

NRI > 0 or IDI > 0 means the parameter improve the predictive value of disease.

*P < 0.05.

**P < 0.001.

NRI, net reclassification improvement; IDI, integrated discrimination improvement; TC, total cholesterol; TG, triglyceride; HDL-C, high-density lipoprotein cholesterol; Apo A-I, apolipoprotein A-I; Apo B, apolipoprotein B; Lp (a), lipoprotein(a); CAD, coronary artery disease.

### Association between Lp (a) and multi-vessel CAD

**FIGURE 3:**
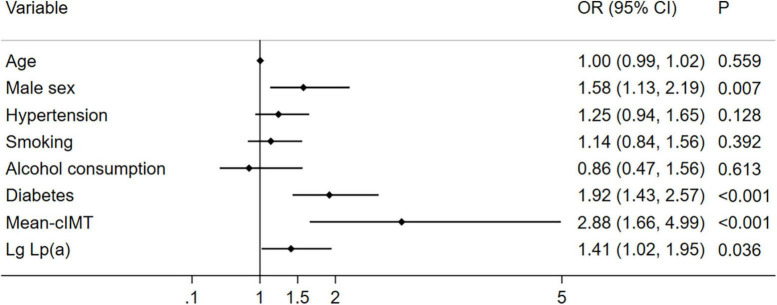
Relationship between Lp (a) combined with cIMT and multi-vessel CAD (*n* = 1004). Illustration: Lp (a) was transferred to Lg Lp (a).

As shown in Figure 3, after adjusting for other risk factors in the CAD group (*n* = 1004), high levels of Lp (a) was associated with multi-vessel CAD than those with low levels of Lp (a) (OR 1.41, 95% CI 1.02–1.95, *P* = 0.036).

## Discussion

By comparing the predictive value of different blood lipid parameters combined with cIMT on CAD, the study had three major findings: (1) Lp (a) better improved the prediction accuracy of CAD in comparison with other blood lipid parameters; (2) in the absence of high levels of LDL-C, the predictive value of Lp (a) on CAD was highlighted; (3) Lp (a) can also be used to predict multi-vessel CAD.

Atherosclerosis is a systemic vascular disease caused by a lipid metabolism disorder and most likely involves the carotid and coronary arteries (29). Although coronary angiography is the gold standard for the diagnosis of CAD, it is an invasive examination that is unsuitable for general screening of CAD. In addition to identifying traditional risk factors such as demographic information and healthy behavior to predict CAD, screening for subclinical atherosclerosis is also important. Carotid ultrasound is a non-invasive examination (18). cIMT is widely recognized as a marker of early stage atherosclerosis and the severity of CAD, which can be used to indirectly assess coronary artery conditions (19, 30). Many studies have shown that cIMT is associated with cardiovascular risk factors and cardiovascular disease (CVD) (31, 32). Furthermore, several cohort studies have found that increased cIMT is associated with future cardiovascular events (33, 34). However, the results of a meta-analysis indicated that the sensitivity and specificity of cIMT for the diagnosis of CAD were 0.68 (95% CI: 0.57–0.77) and 0.70 (95% CI: 0.64–0.75), respectively, which indicated that only cIMT has limited effectiveness as a diagnostic tool for CAD screening (19). In addition, since lipid metabolism disorders cannot be reflected directly, it is of limited value in the use of single cIMT in actual clinical application (21, 35). Combining cIMT and lipid parameters could further improve the diagnostic efficiency of CAD. In recent years, research progress regarding lipid abnormalities has focused on the blood lipid indices, which can reflect lipid metabolism disorders better than a single one (36–38). Wu et al found that multivariate logistic regression analysis showed that Apo B/Apo A-I ratio, a composite index, had a larger OR value than a single index (LDL-C or Apo B) and was better than a single lipid index in the prediction of CAD (36). Similarly, in a cohort study by Rabizadeh et al. the analysis showed that LDL-C/Apo B ratio ≤ 1.2 can independently predict CAD (OR = 1.841, *P* = 0.002) (37). In a cohort study conducted by Kappelle et al. Apo B/Apo A-I ratio and TC/HDL-C ratio were able to predict CAD and the first major adverse event during follow-up (38). Consistent with previous studies, this study found that Lp (a), TC/HDL-C, LDL-C/HDL-C, Apo B/Apo AI, TG/HDL-C, and LDL-C/Apo B ratios were significantly associated with CAD. In terms of effectively predicting CAD, the addition of lipid parameters on traditional risk factors model improved the accuracy of the estimates, and Lp (a) was the best.

Many studies have reported that elevated plasma Lp (a) is an independent risk factor for CAD, which mainly participates in the pathophysiological process of CAD *via* prothrombotic/anti-fibrinolytic effects and promoting the deposition of cholesterol in the vascular intima (39–41). On an equimolar basis, Lp (a) is more likely to cause atherosclerosis than LDL because it not only contains all proatherogenic components of LDL-C but also those of Apo (a) (13). Because the structure of Lp (a) has one more Apo (a) than that of LDL-C, its ability to enhance atherosclerotic thrombosis through other mechanisms including inflammation is stronger than that of LDL-C (42). In a chart-controlled study of 143,087 subjects, Lp (a) concentration was associated with the risk of CAD in a dose-dependent manner, with the risk of CAD increasing as the percentile level of Lp (a) increased (43). With the development of genetic technology, the relationship between Lp (a) and CVD has been well elucidated at the genetic level (44). Many large observational and genetic epidemiological studies have shown that high Lp (a) levels and corresponding LPA genotypes increase the risk of CVD (45, 46). In addition, elevated level of LDL-C is strongly associated with the development of CAD (47). The 2019 ESC guidelines also recommend using LDL-C as the primary indicator of lipid-lowering therapy (48). In the baseline information of the study patients, the LDL-C of the CAD group was similar to that of the non-CAD group, possibly because the CAD group took more statins. In such condition, the levels of LDL-C were comparable in both groups, and the additional effect of Lp (a) could be investigated. Previous studies have shown that patients still have a certain risk of CAD after the treatment for reducing LDL-C, and this residual risk was related to elevated Lp (a) (49–53). In our study, the mean LDL-C level in CAD group was 2.6 mmol/L, meeting the LDL-C management target at a low level. Among patients whose target blood lipids are normal or below the target, Lp (a) will play an important role in the prediction of CAD. This may explain the results that the AUC value of Lp (a) was greater than that of LDL-C-associated blood lipid indices in the ROC curve.

In addition, elevated Lp (a) levels were correlated with multi-vessel CAD. This result is consistent with a recent finding demonstrating that high Lp (a) was significantly associated with increased CAD severity, evaluated using the SYNTAX score (44). The findings of the present study highlight the importance of Lp (a) combined with cIMT in assessing the risk of CAD, especially for those with normal level of LDL-C, which may be useful for guiding primary prevention decisions.

This study has some strengths. This study compared the effectiveness of different lipid parameters combined with cIMT in predicting CAD in the absence of high LDL-C for the first time, and recognized that Lp (a) was superior to other lipid parameters in CAD discrimination, through NRI and IDI. This study has certain limitations. First, this was a retrospective study and no causal conclusion can be drawn. Second, Some people with CAD have a history of statin use, which may have an effect on Lp (a) levels. Third, this study lacked height and weight data to calculate individual body mass index (BMI). As the aim of our study was to compare the predictive value of different lipid parameters rather than to build a predictive model for CAD, the effect of BMI on the primary outcome of this study was not significant. Finally, the severity of CAD was based on the number of coronary artery lesions, and the corresponding degree of stenosis in each branch was not well quantified.

## Conclusion

For patients with highly suspected CAD, Lp (a) combined with cIMT better improved the predictive value of CAD compared with other blood lipid parameters, especially in the absence of high levels of LDL-C. High concentration of Lp (a) was also associated with the multi-vessel CAD. In the future, Lp (a) may need more attention and management in the prevention of CAD.

## Data availability statement

The raw data supporting the conclusions of this article will be made available by the authors, without undue reservation.

## Ethics statement

The studies involving human participants were reviewed and approved by the Ethics Committee of Guangdong Provincial People’s Hospital. The patients/participants provided their written informed consent to participate in this study.

## Author contributions

BY and YW: manuscript preparation and writing—original draft. WL, LZ, and YL: data collection and collation. BY, YW, and WW: data analysis. GL and YZ: writing—critical revisions. XH and XL: conceptualization and approval of the final version of the manuscript for submission. All authors read and approved the final manuscript.
